# *Rickettsia parkeri* as a Probable Agent of Mild Spotted-Fever Group Rickettsiosis Identified by Seroreactivity in Villeta, Colombia

**DOI:** 10.3390/tropicalmed11060164

**Published:** 2026-06-18

**Authors:** Carlos Ramiro Silva-Ramos, Peter C. Melby, Patricia V. Aguilar, Miguel M. Cabada, Juan David Rodas, Marylin Hidalgo, Álvaro A. Faccini-Martínez

**Affiliations:** 1 Grupo de Enfermedades Infecciosas, Departamento de Microbiología, Facultad de Ciencias, Pontificia Universidad Javeriana, Bogotá 110311, Colombia; 2 Department of Pathology, University of Texas Medical Branch, Galveston, TX 77555, USA; 3 Grupo de Investigación Salud TI_Udistrital, Facultad de Ciencias de la Salud, Universidad Distrital Francisco José de Caldas, Bogotá 110711, Colombia; 4 Division of Infectious Diseases, Department of Internal Medicine, University of Texas Medical Branch, Galveston, TX 77555, USA; 5 Center for Tropical Diseases, University of Texas Medical Branch, Galveston, TX 77555, USA; 6 Grupo de Investigación en Ciencias Veterinarias Centauro, Universidad de Antioquia, Medellín 050010, Colombia; 7 Servicio de Infectología, Hospital Militar Central, Bogotá 110231, Colombia; 8 Facultad de Medicina, Universidad Militar Nueva Granada, Bogotá 110231, Colombia

**Keywords:** *Rickettsia parkeri*, spotted fever group, rickettsiosis, differential seroreactivity, acute undifferentiated febrile illness, zoonosis, tick-borne diseases, Colombia

## Abstract

Spotted fever group (SFG) rickettsioses are emerging zoonotic diseases of increasing relevance in Latin America, yet the specific species involved in human infections remain poorly defined in many endemic regions. This study aimed to determine the most probable antigen among SFG-seroreactive febrile patients from Villeta, Colombia. A panel of 25 convalescent-phase serum samples previously identified as positive for SFG *Rickettsia* spp. antibodies was analyzed by indirect immunofluorescence assay using antigens of *Rickettsia rickettsii*, *R. amblyommatis* and *R. parkeri*. Antibody titers were compared to identify differential seroreactivity patterns. Overall, 44% (11/25) of the samples showed differential antibody titers against one of the tested antigens. Among these, nine (36%) exhibited higher titers to *R. parkeri* and two (8%) to *R. amblyommatis*, while none showed exclusive reactivity to *R. rickettsii*. The remaining 56% (14/25) presented similar titers across antigens, consistent with indeterminate or cross-reactive SFG responses. Antibody titers ranged from 1:128 to 1:4096, with *R. parkeri* showing the strongest reactivity. These findings suggest *R. parkeri* or a highly related *Rickettsia* species as the predominant probable antigen in Villeta, highlighting its potential role in mild rickettsial infections and emphasizing the need for eco-epidemiological studies to identify local vectors and reservoirs.

## 1. Introduction

*Rickettsia* (order Rickettsiales, family Rickettsiaceae) is a genus of small, Gram-negative, obligately intracellular bacteria primarily infecting the endothelium, with at least seventeen species recognized as human pathogens [1,2]. Phylogenetically, *Rickettsia* spp. has been classified into several major lineages, commonly including the spotted fever group (SFG), the typhus group (TG), the transitional group (TRG) and the ancestral group (AG). Among these, the SFG and TG comprise multiple species associated with human disease, including *Rickettsia rickettsii*, *R. typhi*, and *R. prowazekii* which have historically been considered clinically significant pathogens [3,4].

Pathogenic *Rickettsia* species cause rickettsioses, a significant and often underestimated cause of zoonotic acute undifferentiated febrile illness (AUFI) worldwide [5]. SFG rickettsioses are tick-borne AUFIs, usually accompanied by macular or maculopapular rash, sometimes with a single or multiple inoculation eschar at the tick bite site [4,6]. Clinical severity varies by species, with *R. rickettsii* being the most virulent, causing severe multisystem damage [4,6].

In the Americas, several pathogenic *Rickettsia* species have been documented with variable clinical severity and epidemiological patterns. *R. rickettsii*, the etiologic agent of Rocky Mountain spotted fever, is widely distributed from Canada to Argentina [1,6]. *Rickettsia parkeri* has emerged over the last two decades as an important cause of milder spotted fever rickettsiosis in North, Central, and South America [1,4]. Other SFG species, including *Rickettsia amblyommatis*, have been detected in ticks throughout the continent, although their role in human disease varies and, in some cases, remains incompletely defined [1,4]. Additionally, *R. typhi*, transmitted by fleas, causes murine typhus in urban and coastal settings in several countries of the Americas [1,4]. This diversity of pathogenic and potentially pathogenic *Rickettsia* species highlights the complex eco-epidemiology of rickettsioses across the continent.

In Colombia, between 1902 and 1986, historical records of SFG and TG rickettsioses, as well as non-specific rickettsial infections, were documented across multiple regions [7]. In the last two decades, at least five endemic areas have been identified: the Tobia Valley in Cundinamarca department, northern Caldas, the Urabá Gulf in Antioquia department, the Caribbean region of Córdoba department, and the eastern plains in the Orinoquia region [8].

In Cundinamarca, the first SFG rickettsiosis cases due to *R. rickettsii* were reported in 1935 during an epidemic in Tobia municipality, with 65 cases identified and 95% mortality [9], with two additional fatal cases recorded in 1941 in neighboring areas [10]. After decades of epidemiological silence, two fatal cases were identified in 2003 and 2004 in Villeta municipality and formally reported in 2007 [11], and a later surveillance identified fifteen non-fatal cases in the same region [12]. These findings establish Tobia Valley and surrounding regions, such as Villeta, as critical regions for active *R. rickettsii* circulation.

Villeta municipality has been the site of several studies focusing on few zoonotic AUFI etiologies, including SFG rickettsioses. In the region, cases of rickettsioses have been reported [11,12], and seropositivity against SFG *Rickettsia* spp. has been documented in both febrile patients and apparently healthy individuals [13]. Additionally, entomological surveys identified *Amblyomma patinoi* ticks as vectors of *R. rickettsii* in the area [14]. Furthermore, serological evidence retrieved from domestic animals, such as dogs and horses, suggests their involvement in local transmission cycles [15].

While the high fatality of *R. rickettsii* in Villeta municipality warrants careful clinical management of AUFI local cases, the occurrence of mild non-fatal SFG rickettsioses cases indicates possible circulation of less virulent human pathogenic SFG species. In a previous study, non-fatal SFG rickettsioses cases were identified including seven cases with seroconversion [16]; however, the specific etiological agent was not determined. Therefore, the aim of the present study was to characterize differential seroreactivity patterns against selected SFG *Rickettsia* antigens in these previously identified mild cases in order to explore the most probable antigen likely associated with prior exposure in this population.

## 2. Materials and Methods

**Figure 1:**
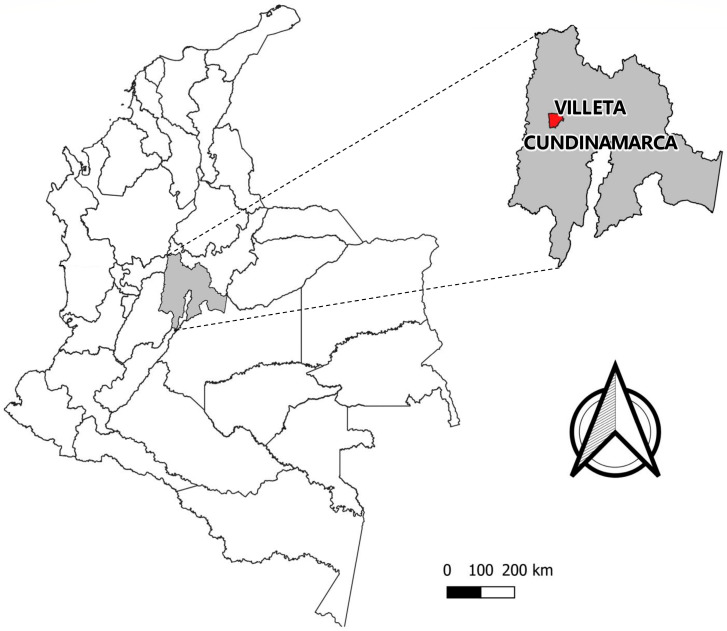
Map showing the location of Villeta municipality (highlighted in red) within Cundinamarca department (highlighted in gray).

Villeta (Figure 1) (5°00′46″ N, 74°28′23″ W) is a municipality in the Gualivá Province, Cundinamarca Department, located 84 km from Bogotá D.C. at 850 m above sea level. The area covers 140 km^2^, includes 22 villages and has a tropical climate with a mean annual temperature of 26 °C and relative humidity between 80% and 97%. Its economy relies mainly on eco-tourism and agriculture, particularly sugarcane and “panela” production. According to the 2018 national census, Villeta has 25,957 inhabitants, of which 17,751 live in the urban area, 743 in suburban settlements and 7463 in rural areas (https://www.dane.gov.co, accessed on 19 December 2025).

Active surveillance was conducted from September to December 2021 at the Salazar de Villeta Hospital as part of a multisite study on AUFI across four countries under a standardized protocol [17]. Patients with a febrile illness lasting less than seven days and without an evident focus of infection were enrolled after clinical evaluation at the emergency department. Serum samples were collected at enrollment (acute phase) and two to three weeks later (convalescent phase), and stored at the “Laboratorio de Bacteriología Especial” of the Pontificia Universidad Javeriana in Bogotá D.C., Colombia.

The study protocol, informed consent, and assent forms were reviewed and approved by the Ethics Committee of the Faculty of Sciences, Pontificia Universidad Javeriana, on 23 May 2019. Written informed consent was obtained from all participants or their legal guardians, and written assent was additionally obtained from minors aged 6 to 18 years. All participants or their legal guardians authorized the storage and future use of their biological samples for research on microorganisms within the scope of the main research project, which included *Rickettsia* species. All samples and records were de-identified using alphanumeric codes. The Ethics Committee of the Faculty of Sciences, Pontificia Universidad Javeriana, does not assign specific numerical approval codes to approved protocols. The study adhered to the ethical regulations established in Resolution 8430 of 1993 of the Ministry of Health of Colombia and to the principles of the Declaration of Helsinki.

Serum samples that previously tested positive for SFG rickettsiosis in a prior study, based on evidence of seroconversion or antibody titers ≥ 1:256 [16], were selected for further analysis. Additional samples reactive at a 1:128 dilution within the same cohort were also included. These samples were subsequently analyzed by indirect immunofluorescence assay (IFA) to identify the most probable SFG antigen associated with the observed serological response. In-house IFA testing was performed using pre-prepared antigen-coated slides containing three SFG *Rickettsia* species: *R. rickettsii* strain Sheila Smith, *R. parkeri* strain Atlantic Rainforest, and *R. amblyommatis* isolated from Texas, United States. All antigen-coated slides were prepared and supplied by Lucas S. Blanton from the University of Texas Medical Branch (UTMB), Galveston, Texas, United States under a Material Transfer Agreement. Antigen preparation, bacterial cultivation and standardization of antigen concentration were performed at UTMB following previously established protocols [18]. Upon receipt, slides were stored at −80 °C in vacuum-sealed containers until use to preserve antigen integrity and prevent ice crystal formation. The selection of these SFG *Rickettsia* species was guided by epidemiological reports indicating the circulation of these infectious agents in the region and elsewhere in Colombia.

Serological evaluation was performed using only convalescent-phase serum samples obtained from SFG-seropositive febrile patients. Antigen-coated slides were initially equilibrated in phosphate-buffered saline (PBS) 1X for 10 min, followed by incubation in PBS 1X supplemented with 1% bovine serum albumin (BSA) for 15 min. Slides were then allowed to air-dry at room temperature prior to serum application. Each serum sample was initially screened at a dilution of 1:128 in PBS 1X with 1% BSA, which was established as the serological cut-off for all three SFG antigens evaluated. Samples reactive at this dilution were subsequently subjected to antibody titration using two-fold serial dilutions (1:256, 1:512, 1:1024, and higher as required) in PBS 1X until the endpoint titer was reached. The endpoint titer was defined as the highest serum dilution that exhibited specific fluorescence against each antigen tested. For each dilution, 10 μL of diluted serum were added to individual wells of the antigen-coated slides. Slides were incubated at 37 °C for 1 h in a humidified chamber. Following incubation, slides were rinsed once and washed twice for 10 min each with PBS 1X containing 0.1% Tween 20. After washing, wells were incubated with a fluorescein-labeled secondary antibody. Detection of IgG antibodies was performed using Alexa Fluor^®^ 488 AffiniPure™ Donkey Anti-Human IgG Fcγ fragment-specific conjugate (Jackson ImmunoResearch Inc., West Grove, PA, USA) diluted 1:800 in PBS 1X with 1% BSA. Slides were incubated for an additional 1 h at 37 °C and subsequently washed twice with PBS 1X containing 0.01% Tween 20 and 0.001% Evans Blue. Slides were mounted using buffered glycerin under coverslips and examined by fluorescence microscopy at 100x magnification. All assays were performed in duplicate under controlled temperature and humidity conditions. Identical reagents, equipment, incubation times, and light exposure intensities were used throughout slide processing and reading to ensure reproducibility and comparability of results prior to reporting the endpoint antibody titers for each of the three *Rickettsia* spp. antigens evaluated.

To estimate the most probable *Rickettsia* antigen responsible for the observed serological response, antibody titers obtained against the three tested SFG antigens were compared for each serum sample. The most likely antigen was defined as the *Rickettsia* species for which the antibody titer was at least two serial dilutions higher (four-fold increase) than those observed against the other antigens. When antibody titers differed by fewer than two dilutions or were identical across antigens, results were classified as cross-reactive, indicating exposure to an undetermined SFG *Rickettsia* species. The assay included positive and negative control sera characterized prior to testing. Positive control sera consisted of samples previously confirmed by seroconversion using IFA in combination with isolation of *R. rickettsii* [19], *R. parkeri* [20], and *R. amblyommatis* (unpublished data) from human individuals, each displaying antigen-specific reactivity with at least a two-fold higher titer. Negative control sera were obtained from individuals previously characterized as seronegative using the same reference methods, as described in a previous study [16].

## 3. Results

The analysis comprised 25 convalescent-phase serum samples that tested positive for SFG *Rickettsia* spp. antibodies. Among these, 22 had been previously reported as part of the etiological findings of AUFI cases, with seven showing a four-fold seroconversion and fifteen exhibiting antibody titers ≥ 1:256; while the remaining three additional samples had antibody titers of 1:128.

Overall, 44% (11/25) of the evaluated samples showed differential antibody titers against one of the three *Rickettsia* antigens tested. Among these, nine samples (36% [9/25]) showed higher antibody titers against *R. parkeri* antigens, including two cases with seroconversion, while the remaining two samples (8% [2/25]) showed higher titers against *R. amblyommatis* antigens, both without evidence of seroconversion. None of the samples tested demonstrated a serological profile consistent with exclusive exposure to *R. rickettsii*.

The remaining samples (56% [14/25]) displayed similar titers for all the antigens tested, with no substantial differences beyond the established cut-off point. These were classified as samples with indeterminate seroreactivity or cross-reactivity responses within the SFG. Positive controls using serum samples from patients confirmed with *R. rickettsii*, *R. parkeri*, and *R. amblyommatis* infections showed specific reactivity for their respective homologous antigens. Negative controls showed no evidence of seroreactivity at the screening dilution of 1:128 against any of the evaluated antigens. Detailed information on each serum sample and its corresponding titers against the three antigens tested can be observed in Table 1.

Antibody titers ranged from 1:128 to 1:4096, showing variable reactivity patterns among the seropositive samples. Among samples with differential seroreactivity, the highest antibody titers were observed against *R. parkeri* antigens, reaching up to 1:4096, whereas reactivity against *R. amblyommatis* antigens reached titers of up to 1:2048. Taken together, these findings indicate that, although most samples showed indeterminate seroreactivity, *R. parkeri* was the predominant most probable antigen among samples with differential serological responses, followed by *R. amblyommatis*.

## 4. Discussion

Eleven of the analyzed samples exhibited differential antibody titers against the evaluated antigens, of which, nine showed greater reactivity against *R. parkeri*. Rickettsiosis due to *R. parkeri* is currently recognized as the second most prevalent SFG rickettsiosis in the Americas [21,22,23]. Clinically, it manifests as a mild AUFI, often with a favorable prognosis, and commonly associated with the presence of an inoculation eschar at the tick bite site [23]. Villeta and its surrounding areas are recognized as one of the main endemic regions for rickettsiosis in Colombia, where the circulation of *R. rickettsii*, transmitted by *Amblyomma patinoi* ticks, has been documented for several decades [9,11,14]. Historically, SFG rickettsiosis outbreaks in Villeta were characterized by extremely high lethality, reaching 95% mortality, with only three survivors among 65 identified cases, representing one of the highest fatality rates ever reported in the scientific literature [9]. Although fatal cases attributed to *R. rickettsii* were reported again decades later [11], subsequent studies documented milder and non-fatal cases of SFG rickettsioses [12]. These findings raised the hypothesis that other, less virulent SFG *Rickettsia* species might be circulating in the region.

The serological evidence obtained in the present study is compatible with exposure to *R. parkeri* or a closely related SFG *Rickettsia* species within this cohort, which is epidemiologically plausible given its previous identification in another region from Colombia associated with AUFI [20,24]. The detection of differential seroreactivity is consistent with possible exposure to *R. parkeri* within the analyzed cohort. Although *R. rickettsii* has historically been the primary focus of surveillance due to its high lethality, the possible presence of *R. parkeri* or a related SFG species could contribute to the occurrence of milder, non-fatal cases of SFG rickettsiosis recently reported in the region. Additionally, although *R. parkeri* has been detected in association with wild birds and their ectoparasitic ticks in other regions of Colombia, these findings are mainly linked to the non-pathogenic NOD strain [24,25]. In contrast, if present, the epidemiological cycle of *R. parkeri* in Villeta would most plausibly be associated with *Amblyomma* spp., particularly *A. ovale* or species within the *A. maculatum* complex, which are recognized vectors of pathogenic *R. parkeri* strains, including *R. parkeri* sensu stricto and the Atlantic rainforest strain. The presence of these ticks in peri-domestic and rural environments could facilitate human exposure and sporadic spotted fever-like illness in the region. Consequently, these findings highlight the need for future studies aiming to identify the specific tick vectors and potential vertebrate reservoirs involved in the local transmission cycle of this emerging *Rickettsia* species in Villeta municipality.

Additionally, two of the analyzed serum samples showed differential reactivity against *R. amblyommatis*, a species widely distributed throughout the Americas that infects multiple tick species, primarily *Amblyomma* [26]. However, despite its broad geographic distribution and range of tick hosts, *R. amblyommatis* has not been conclusively linked to human disease. In the scientific literature, two case reports described imported ticks in which molecular analyses detected only *R. amblyommatis*; however, despite vector exposure, both individuals experienced either vague, non-specific symptoms or no clinical manifestations at all [27,28]. Bioinformatics studies have demonstrated that this *Rickettsia* species retains several virulence-associated genes related to SFG rickettsioses pathogenesis, but exhibits defects in endothelial adhesion and slower replication, suggesting that high infectious doses would be required to cause disease [29]. Experimental murine models have shown minimal or even undetectable histopathological lesions following *R. amblyommatis* infection [30], supporting the hyphotesis that most human infections are subclinical or, rarely, very mild. Accordingly, prior exposure to *R. amblyommatis* may induce partial protective immunity against more virulent species such as *R. rickettsii*, highlighting its potential as an attenuated vaccine candidate [31]. Therefore, it is plausible that exposure to *R. amblyommatis* in Villeta may have provided the local population with partial immune protection, which could help explain the absence of recent fatal cases of SFG rickettsiosis due to *R. rickettsii* in this historically hyperendemic area; however, this hypothesis requires further investigation.

Notably, none of the analyzed samples displayed a serological profile compatible with exclusive exposure to *R. rickettsii*, despite the known active circulation of this pathogen in the region [11,14]. This finding may indicate that other, less-studied SFG *Rickettsia* spp. could also be contributing to rickettsial infections within this cohort in Villeta municipality. Furthermore, fourteen of the tested serum samples did not show exclusive reactivity against any of the evaluated antigens, implying potential exposure to other *Rickettsia* species not included in the present analysis. Such diversity suggests complex eco-epidemiological dynamics of Rickettsia spp. within this endemic setting, as well as in other largely unexplored areas of Colombia where these dynamics are likely underestimated, as rickettsioses are not yet included among the compulsory notifiable diseases within the national surveillance system [32], despite the substantial burden of disease they impose.

Importantly, seroconversion was observed in only seven of the twenty-five paired serum samples [16], indicating that most AUFI cases were unlikely to represent acute rickettsial infection. Although nine of twenty-five patients exhibited greater seroreactivity against *R. parkeri*, only two of them demonstrated seroconversion, supporting recent infection. The remaining seven patients with reactivity against *R. parkeri* did not show antibody titer increases between acute and convalescent samples and therefore most likely represent previous exposure rather than active disease. In a historically endemic region such as Villeta, background IgG seropositivity is epidemiologically plausible due to repeated exposure to infected ticks. Thus, while acute rickettsial infection was confirmed in a subset of cases, the overall seropositivity observed also reflects prior circulation of SFG *Rickettsia* species in the local population.

Interestingly, the presence of rash showed no clear association with a specific most probable *Rickettsia* antigen based on differential seroreactivity. However, a notable correlation was observed between rash and seroconversion. Among the seven patients who demonstrated seroconversion, five presented with rash. Conversely, five of the seven patients with rash exhibited seroconversion. Although eschar is considered an important clinical finding in some SFG rickettsioses such as the one caused by *R. parkeri*, its presence was not systematically assessed as part of the original study protocol and therefore could not be evaluated in this analysis.

## 5. Conclusions

Serological analysis of SFG-seroreactive samples from febrile patients in Villeta identified *R. parkeri* (or a closely related SFG *Rickettsia* species) as the most probable antigen within this cohort among individuals with differential seroreactivity. Although IFA does not allow definitive species-level attribution, the observed differential seroreactivity patterns provide epidemiological evidence suggesting the circulation of SFG *Rickettsia* species other than *R. rickettsii*, which is known to be endemic in the study area. The absence of exclusive reactivity against *R. rickettsii* and the presence of indeterminate or cross-reactive responses in over half of the samples further support the complexity of SFG rickettsial exposure within the analyzed population. These findings should be interpreted cautiously and do not establish definitive etiological attribution in the absence of cross-absorption studies, molecular confirmation or microbial isolation. However, they support the possibility that *R. parkeri* or another closely related SFG species may contribute to mild rickettsial presentations in Villeta and highlight the need for additional studies using molecular and isolation-based approaches to better characterize the diversity of circulating rickettsiae in the region. In addition, surveillance of AUFI cases presenting with an inoculation eschar and a history of tick exposure should systematically be performed, as this clinical sign is a hallmark of *R. parkeri* rickettsiosis and may facilitate timely clinical suspicion in endemic settings.

## Figures and Tables

**Table 1:** Comparison of antibody titers to *Rickettsia rickettsii*, *R. amblyommatis* and *R. parkeri* in analyzed convalescent-phase serum samples and identification of the most probable antigen.

Sample ID	Result from Silva-Ramos et al. 2023 [16]		*R. rickettsii* Titration	*R. amblyommatis* Titration	*R. parkeri* Titration	Most Probable Antigen	Presence of Rash
	Acute Phase Titer	Convalescent Phase Titer					
COV001	1:512	1:1024	1:1024	1:1024	1:4096	*R. parkeri*	No
COV003	1:128	1:128	1:128	1:128	1:512	*R. parkeri*	No
COV004	1:256	1:512	1:512	1:512	1:512	Undetermined	Macular
COV007 *	Negative	1:256	1:256	1:256	1:256	Undetermined	Macular
COV009	1:1024	1:2048	1:2048	1:2048	1:2048	Undetermined	No
COV013	1:512	1:512	1:512	1:512	1:512	Undetermined	No
COV016	1:128	1:256	1:256	1:256	1:256	Undetermined	No
COV022 *	Negative	1:512	1:512	1:512	1:512	Undetermined	No
COV025	1:256	1:512	1:512	1:512	1:2048	*R. parkeri*	No
COV026 *	1:512	1:2048	1:2048	1:2048	1:2048	Undetermined	No
COV030 *	Negative	1:512	1:512	1:512	1:2048	*R. parkeri*	Macular
COV032	1:1024	1:1024	1:1024	1:1024	1:1024	Undetermined	No
COV034	1:128	1:128	1:128	1:256	1:1024	*R. parkeri*	No
COV035	1:512	1:512	1:512	1:2048	1:512	*R. amblyommatis*	No
COV037 *	1:256	1:1024	1:1024	1:1024	1:1024	Undetermined	Macular
COV038	1:256	1:512	1:512	1:512	1:2048	*R. parkeri*	No
COV040	1:256	1:512	1:512	1:512	1:2048	*R. parkeri*	No
COV041 *	1:256	1:1024	1:1024	1:1024	1:2048	Undetermined	Macular
COV042	1:256	1:512	1:512	1:1024	1:1024	Undetermined	No
COV043 *	Negative	1:256	1:256	1:256	1:1024	*R. parkeri*	Macular
COV044	1:512	1:1024	1:1024	1:1024	1:1024	Undetermined	Macular
COV045	1:128	1:128	1:256	1:256	1:1024	*R. parkeri*	No
COV047	1:256	1:512	1:512	1:1024	1:1024	Undetermined	No
COV053	1:256	1:512	1:512	1:512	1:512	Undetermined	No
COV056	1:256	1:256	1:256	1:1024	1:256	*R. amblyommatis*	No
*R. rickettsii* positive control	N/A	N/A	1:4096	1:1024	1:1024	*R. rickettsii*	N/A
*R. amblyommatis* positive control	N/A	N/A	1:128	1:512	1:128	*R. amblyommatis*	N/A
*R. parkeri* positive control	N/A	N/A	1:128	1:256	1:1024	*R. parkeri*	N/A

* Evidence of seroconversion.

## Data Availability

The raw data supporting the conclusions of this article will be made available by the authors on request.
